# Supplementary material for: Mycobacterium avium subsp. paratuberculosis MAP1889c Protein Induces Maturation of Dendritic Cells and Drives Th2-Biased Immune Responses
Source: Cells. 2020 Apr 11;9(4):944. doi: 10.3390/cells9040944 (PMC7226993; doi:10.3390/cells9040944)
Supplement: Supplementary file 1 [file cells-09-00944-s001.pdf]

Supplementary figures

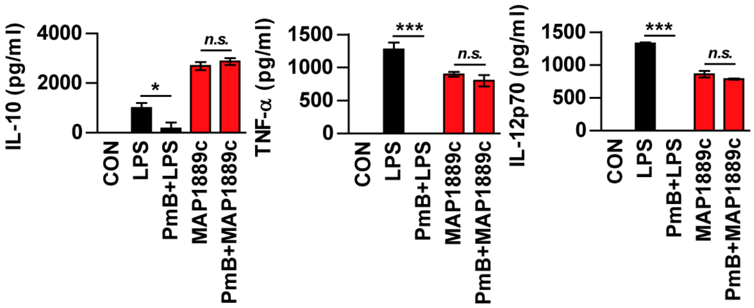

Figure S1. Confirmation of endotoxin decontamination of the purified MAP1889c. BMDCs were incubated with LPS (100 ng/mL) or MAP1889c (10 μg/mL) with or without pretreatment with polymyxin B (PmB) for 1 h. After 24 h, IL-10, TNF-α, and IL-12p70 production in the culture supernatants was analyzed by ELISA. All data are expressed as the mean ± SD ($n = 3$). * $p < 0.05$, and *** $p < 0.001$ for treatment compared with the difference between treatment data. *n.s.*, no significant difference.

A

Dye	Unstained control	FMO control	Sample
BV605	-	CD4	CD4
APC-cy7	-	CD8	CD8
BV510	-	-	IL-10

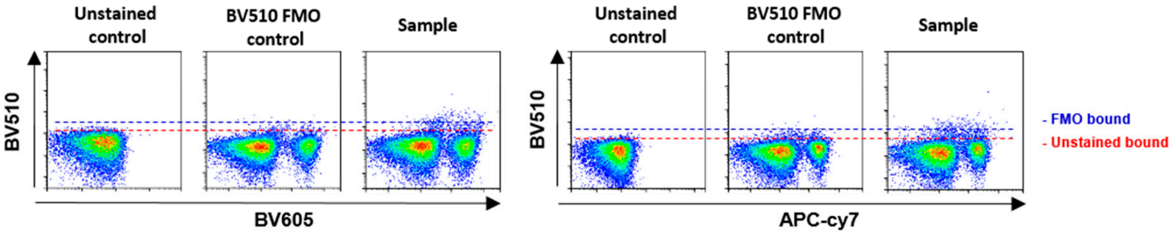

B

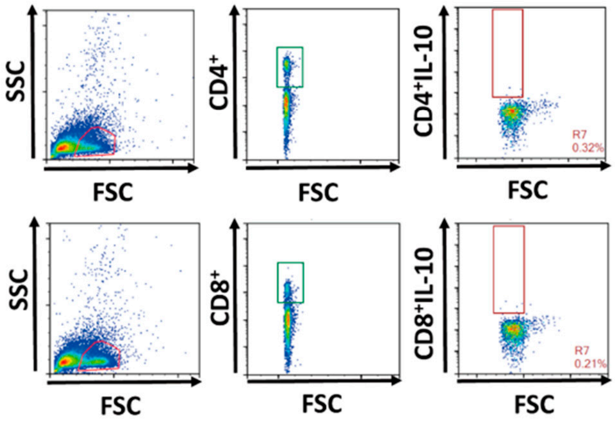

Figure S2. Gating strategy for IL-10 cytokine from T cells for Figure 6E. (**A**) FMO control performed by excluding only the flour of interest. (**B**) After that, gating was selected based on the FMO bound line. Next, each of the CD4⁺ and CD8⁺ cells was selected. For cells in each of the CD4⁺ and CD8⁺ gates for IL-10 were then established.

A

Dye	Unstained control	FMO control	Sample
BV605	-	CD4	CD4
APC-cy7	-	CD8	CD8
Alexa 488	-	-	T-bet
PE-cy7	-	-	GATA-3

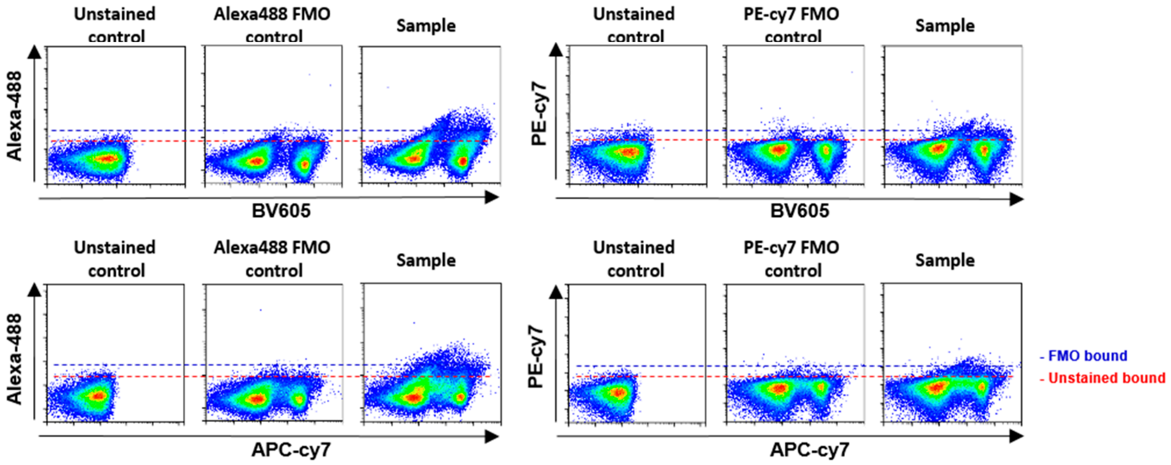

B

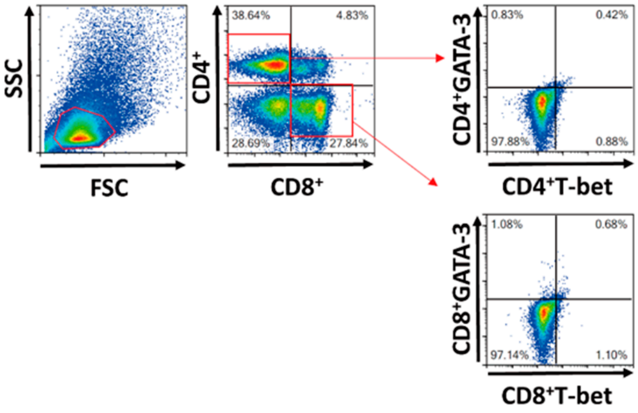

Figure S3. Gating for transcription factor T-bet and GATA-3 from T cells for Figure 7B. (**A**) FMO control by excluding only the flour of interest. (**B**) After that, gating was selected based on the FMO bound line. Next, each of the $CD4^+$ and $CD8^+$ cells was selected. For cells in each of the $CD4^+$ and $CD8^+$ gates, individual gates for T-bet and GATA-3 were then established.

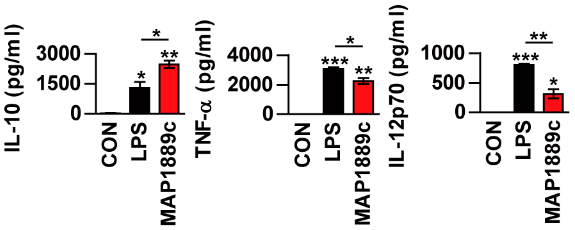

Figure S4. MAP1889c induces high levels of IL-10 production in BMDMs. The BMDMs were stimulated with MAP1889c (10 μg/mL), LPS (100 ng/mL) for 24 h. IL-10, TNF-α, and IL-12p70 cytokines from the culture supernatants were measured by ELISA.
